# Overexpression of *SQUALENE SYNTHASE* Reduces *Nicotiana benthamiana* Resistance against *Phytophthora infestans*

**DOI:** 10.3390/metabo13020261

**Published:** 2023-02-11

**Authors:** Ke-Ke Fu, Junhao Liang, Wei Wan, Xiangfeng Jing, Hongjie Feng, Yanling Cai, Shaoqun Zhou

**Affiliations:** 1 Zhengzhou Research Base, State Key Laboratory of Cotton Biology, School of Agricultural Sciences, Zhengzhou University, Zhengzhou 450001, China; 2 Shenzhen Branch, Guangdong Laboratory of Lingnan Modern Agriculture, Key Laboratory of Synthetic Biology, Ministry of Agriculture and Rural Affairs, Agricultural Genomics Institute at Shenzhen, Chinese Academy of Agricultural Sciences, Shenzhen 518120, China; 3 Key Laboratory of Northwest Loess Plateau Crop Pest Management of Ministry of Agriculture and Rural Affairs, Northwest A&F University, Yangling 712100, China; 4 State Key Laboratory of Silkworm Genome Biology, Southwest University, Beibei, Chongqing 400715, China

**Keywords:** potato late blight, tomato late blight quantitative disease resistance, triterpenoids, phytosterols

## Abstract

Plant triterpenoids play a critical role in plant resistance against *Phytophthora infestans* de Bary, the causal pathogen of potato and tomato late blight. However, different triterpenoids could have contrasting functions on plant resistance against *P. infestans*. In this study, we targeted the key biosynthetic gene of all plant triterpenoids, *SQUALENE SYNTHASE* (*SQS*), to examine the function of this gene in plant–*P. infestans* interactions. A post-inoculation, time-course gene expression analysis revealed that *SQS* expression was induced in *Nicotiana benthamiana* but was transiently suppressed in *Solanum lycopersicum*. Consistent with the host-specific changes in *SQS* expression, concentrations of major triterpenoid compounds were only induced in *S. lycopersicum*. A stable overexpression of *SQS* in *N. benthamiana* reduced plant resistance against *P. infestans* and induced the hyperaccumulation of stigmasterol. A comparative transcriptomics analysis of the transgenic lines showed that diverse plant physiological processes were influenced by *SQS* overexpression, suggesting that phytosterol content regulation may not be the sole mechanism through which *SQS* promotes plant susceptibility towards *P. infestans*. This study provides experimental evidence for the host-specific transcriptional regulation and function of *SQS* in plant interactions with *P. infestans*, offering a novel perspective in examining the quantitative disease resistance against late blight.

## 1. Introduction

*Phytophthora infestans* de Bary is a destructive oomycete pathogen that causes late blight on tomatoes (*Solanum lycopersicum*) and potatoes (*Solanum tuberosum*). As one of the most notorious phytopathogens, *P. infestans*-associated diseases can cost USD 3–10 billion in yield loss and management across the globe [1,2]. Currently, a prompt application of chemical pesticides remains the most effective control measure against *P. infestans*. Over-dependence on the chemical control of *P. infestans* has taken not only a financial toll on potato and tomato production but has also raised environmental and health hazard risks. Due to these concerns, the European Union has recently discontinued the use of pesticides containing mancozeb, one of the most widely adopted active ingredients against *P. infestans*, pressing for safer and more economic strategies for the control of *P. infestans* [3].

The genetic enhancement of crop disease resistance is a promising approach for the sustainable management of late blight. The main efforts in dissecting plant genetic resistance against *P. infestans* in recent decades focused on the identification of dominant resistance (*R*) genes [4,5]. Though *R* genes can provide highly effective protection against incompatible *P. infestans* pathovars, their recognition spectra are limited such that the quickly evolving pathogen populations can escape from the immunity conferred by *R* genes. Dominant *R* genes are supplemented by diverse quantitative disease resistance (QDR) genes, which provide weaker but more persistent disease resistance [6]. A number of QDR genes have been characterized for resistance against *P. infestans*. Functionally, these QDR genes are involved in the biosynthesis, regulation, and transport of plant-specialized metabolites [7,8,9,10,11,12,13,14,15,16,17,18].

Plant-produced triterpenoids are a large group of small molecules that function as structural constituents (e.g., phytosterol), signaling molecules (e.g., brassinosteroids), and defensive, specialized metabolites (e.g., steroidal glycoalkaloids). The diverse functions of different triterpenoid compounds complicate the elucidation of their roles in plant-–*P. infestans* interactions. *Phytophthora* and other oomycetes cannot produce sterols, and plant-derived sterols can stimulate *P. infestans* vegetative growth and sporulation [1,19]. On the other hand, brassinosteroid signaling has been shown to suppress host immunity against *P. infestans* [20,21]. The hallmark steroidal glycoalkaloids of tomatoes and potatoes were unable to inhibit *P. infestans* growth in vitro [22]. More recently, a comparative metabolomics study has revealed that a resistant potato cultivar tended to accumulate higher contents of triterpenoid compounds than a susceptible relative, and that these compounds tended to be induced by *P. infestans* in the susceptible cultivar but were suppressed in the resistant line [23].

**Figure 1:**
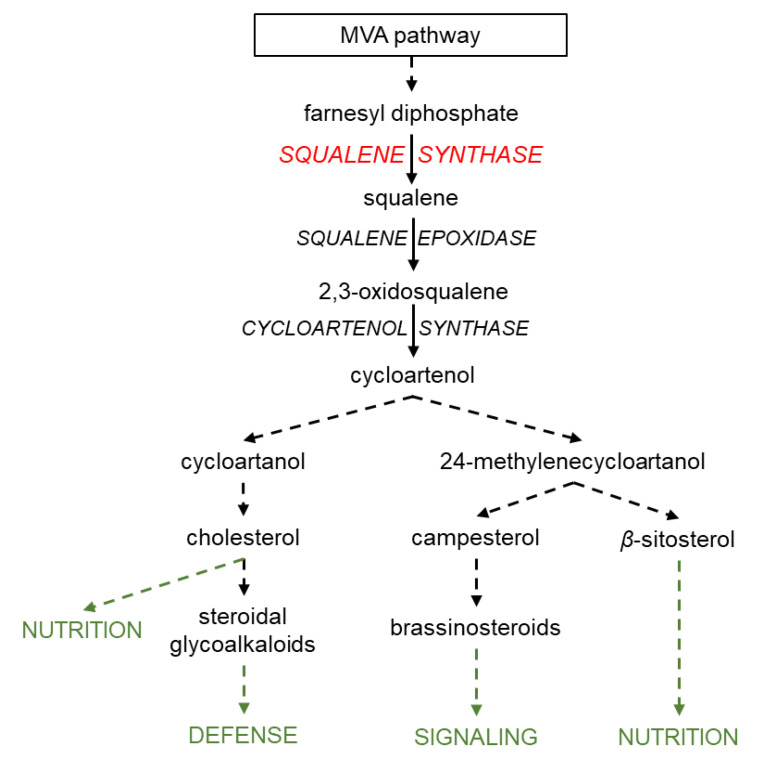
*SQUALENE SYNTHASE* catalyzes the biosynthesis of squalene, the common metabolic precursor of all known plant triterpenoids. Genes encoding metabolic enzymes are in all-capitalized, italicized letters. Dashed arrows indicate multi-step metabolic pathways.

In land plants, the vastly diverse triterpenoids are derived from the common precursor squalene, which provides the fundamental thirty-carbon backbone [24] (Figure 1; De Vriese et al. 2021). This shared biosynthetic origin provides the opportunity to examine the collective effects of triterpenoids on *P. infestans* resistance through genetic manipulation of the common upstream biosynthetic gene, *SQUALENE SYNTHASE* (*SQS*). In this study, we examined the expression of *SQS* and the dynamic accumulation of phytosterols in *Nicotiana benthamiana* and *S. lycopersicum* upon *P. infestans* inoculation. We then generated *SQS*-overexpressing *N. benthamiana* (oeNbSQS) plants and measured their resistance levels against *P. infestans*. Finally, targeted phytosterol measurements and comparative transcriptomics analyses of oeNbSQS and wild type plants shed light on the potential metabolic and transcriptomic alterations associated with enhanced *P. infestans* resistance of these plants.

## 2. Materials and Methods

### 2.1. Growth Condition of Plant Materials

All the plants were grown in individual, 3 inch × 3 inch plots under glasshouse cycle conditions of 16 h of light at 24 °C, 8 h of dark at 20 °C, and a relative humidity kept between 50 and 70%. Approximately four- to five-week-old *Nicotiana benthamiana* plants and six- to eight-week-old tomato cv. Micro-Tom (*Solanum lycopersicum*) plants were used for the experiments.

### 2.2. Phytophthora Infestans Infection Assay

The *P. infestans* strain T30-4 was grown on rye (*Secale cereale*) agar at 18 °C in the dark for 2 weeks. To harvest sporangia, each rye agar plate containing sporangia were soaked with 10 mL of sterilized H_2_O, and sporangia were collected after 3–4 h of incubation at 4 °C. The suspension containing sporangia was spun down at 3000 rpm for 10 min, and the sporangia concentrations were quantified using a hemocytometer. Droplets (10 μL) of a solution of 100,000 zoospores per mL were applied onto the abaxial sides of detached *N. benthamiana* and tomato leaves and incubated on wet paper towels in 100% relative humidity, in the dark, at 18 °C. The lesion diameter was measured 7 d post-inoculation.

For trypan blue staining, the infected plant leaves were soaked in trypan blue solution (20 mg trypan blue (Sigma), 10 mL lactic acid, 10 mL glycerol, 10 mL water-saturated phenol, 10 mL distilled water, and 40 mL 100% ethanol) and heated up to 100 °C for 5 min. The stained leaves were then transferred into 2.5g/mL chloral hydrate (Sigma) overnight. Pictures were taken when the uninfected area became colorless.

### 2.3. Virus-Induced Gene Silencing

Virus-induced gene silencing (VIGS) was performed using a tobacco rattle virus vector. The VIGS fragment of *NbSQS* (*U46000.1*, GeneBank) was designed with a web-based tool (https://vigs.solgenomics.net) (accessed on 1 July 2020). The fragment was cloned from *N.benthamiana* cDNA and inserted into the pTRV2 vector. The paired vectors were transformed into the *Agrobacterium tumefaciens* strain GV3101. The infiltration buffer, which contained a mixture of pTRV1 and NbSQS-pTRV2 construct at OD600 = 0.5, was infiltrated into the upper leaves of 4-leaf-stage *N. benthamiana* plants. The infiltration buffer contained 10 mM of MgCl_2_, 10 mM of 2-[N-morpholino] ethanesulfonic acid with pH = 5.6, and 200 μM of acetosyringone. The construct GFP-pTRV2 was used as the negative control, and PDS-pTRV2 was used as the positive control. One week after infection, the leaves were detached for total RNA extraction and gene-expression measurement using q-RT-PCR. Primers for the q-RT-PCR were provided in Supplemental Table S1.

### 2.4. Stable Transformation of N. benthamiana

The full length of the *NbSQS* CDS was cloned from *N. benthamiana* cDNA and inserted into the pSUPER1300 plasmid [25]. *Agrobacterium tumefaciens* GV3101, containing the overexpression vector NbSQS-super1300, was used to transform leaf discs of *N. benthamiana*. Transgenic plants were obtained with an established method [26].

### 2.5. Gene Expression Analysis

Young leaves were collected from plants to extract total RNA using the Plant RNA Extraction Kit (Huayueyang, Beijing). The first-strand cDNA was synthesized from 1 mg of RNA using the HiScript^®^ III 1st Strand cDNA Synthesis Kit with a DNA wiper (Vazyme). Q-RT-qPCR reactions were performed using the ChamQ Universal SYBR qPCR Master Mix kit (Vazyme). The *N. benthamiana* gene *EF1a* (*Niben101Scf07423g04011.1*) was used as a reference control. The tomato gene *SlUBI* (*Solyc01g068045.2*, ITAG3.2) was used as a reference control. Gene expression levels were calculated by a comparative method, as described in Applied Biosystems instructions. For transgenic plant verification, plants without exogenous vectors were used as the negative control. The primers are shown in the Supplemental Table S1.

### 2.6. RNA-Seq Analysis

The leaves of wild type plants and the T3 progeny of the *NbSQS*-overexpressing plant were used to extract RNA for a transcriptome analysis. A total of 1 μg of RNA per sample was used for the library construction. The library construction and sequencing were performed by Beijing Novogene Bioinformatics Technology. For each biological sample, six gigabytes of raw reads were obtained. Raw reads in a fastq format were first processed with Perl scripts to obtain clean data. The clean reads were used to calculate the Q20, Q30, and GC contents. The fastq files containing these clean reads were depositedin the NCBI database under PRJNA930498. The reference genome published on the Solanaceae website was downloaded and used to build an index. Paired-end, clean reads were aligned to the reference genome using STAR 2.4.0j software [27]. The read numbers mapped to each gene were counted using Htseq 0.11.1. The fragments per kilobase per million (FPKM) was calculated [28]. A differential expression analysis was performed using the DESeq2 R package (v1.22.2) with an adjusted log2FoldChange > 1 and an FDR < 0.05 [29]. The gene ontology (GO) enrichment analysis was performed using the R package GSEABase and GOstats [30,31]. The background gene ontology (GO) enrichment annotation was re-analyzed by Argot2.5 [32].

### 2.7. Phytosterol Measurement

The method for sterol extraction and quantification was described previously [33]. Briefly, each leaf sample was collected and placed into a 2 mL centrifuge tube. All leaf samples were stored at −20 °C for subsequent analysis. Leaf samples were freeze-dried. Approximately 10 mg of leaf tissue was weighed out from each sample and transferred into a 1.5 mL tube, which contained two zirconia beads (2 mm diameter, 95%). All samples were homogenized at 60 Hz for 2 min (Tissuelyser-24, Shanghai Jingxin, China). To extract the sterols from the tissue, 0.5 mL of methanol and 0.5 mL of chloroform were added into each tube, and 10 µg of cholestane was added into each sample as an internal standard. After a vigorous vortex, 0.45 mL of H_2_O was added to the tube, and the lower layer was transferred into a 20 mL glass vial. To hydrolyze the phytosterol esters, 8 ml of an aqueous, 70% methanol solution containing 5% NaOH was added. The solutions were heated at 60 °C for 2 h, and 3 mL of water was added. Sterols were extracted three times with hexane. The hexane solution was transferred into a new vial and dried in a fume hood. Then, 1 mL of hexane was added to dissolve sterols and transferred into a 2 mL vial, in which 100 µL of 1-(Trimethylsilyl) imidazole (SIGMA, St. Louis, MO, USA) was added to derivatize the sterols. The reaction was terminated by adding 300 µL of 70% methanol/H_2_O and 100 µL of hexane. The hexane part was washed with 70% methanol/H_2_O three times, and was then used for identification and quantification by GC-MS (Thermo Fisher Scientific, Waltham, MA, USA) coupled with a DB-5MS column (Agilent Technologies, Santa Clara, CA, USA). All derivatized sterols were characterized by the derivatized standards. The data were analyzed using Thermo Xcalibur version 2.2 SP1.48 (Thermo Fisher Scientific, Waltham, MA, USA).

### 2.8. Experimental Designs and Statistical Analyses

For the post-inoculation, time-course q-RT-PCR and phytosterol measurement experiments, plant samples were collected from the same batch of plants grown under controlled environmental conditions. Water suspension of *P. infestans* zoospores were drop-inoculated onto the surfaces of detached leaves at a different time, prior to a common harvest time point. This experimental design minimized the potential influence of circadian rhythm on plant gene expression and metabolite abundance. For the control group, sterilized water droplets were used to replace the zoospore suspension and were applied immediately before tissue harvest. All treatment groups were individually compared to the common control group, with one-tailed Student’s *t*-tests assuming equal variance.

For phenotypic, metabolic, and transcriptomic characterizations of the oeNbSQS plants, the transgenic plants were simultaneously planted with their wild type progenitor, and the plants were grown in the same growth chamber under the conditions described above. After one month of growth, the third expanded leaves of each plant were harvested into liquid nitrogen at the same time and ground into a fine powder. Each biological sample was divided into two aliquots for phytosterol measurements and RNA-seq analysis. Zoospore inoculation bioassays were carried out as described above. Post-inoculation lesion diameters and phytosterol measurement data were compared between the overexpression and wild type plants, with one-tailed Student’s *t*-tests assuming equal variance.

## 3. Results

### 3.1. SQUALENE SYNTHASE Expression Is Induced by P. infestans Infection

**Figure 2:**
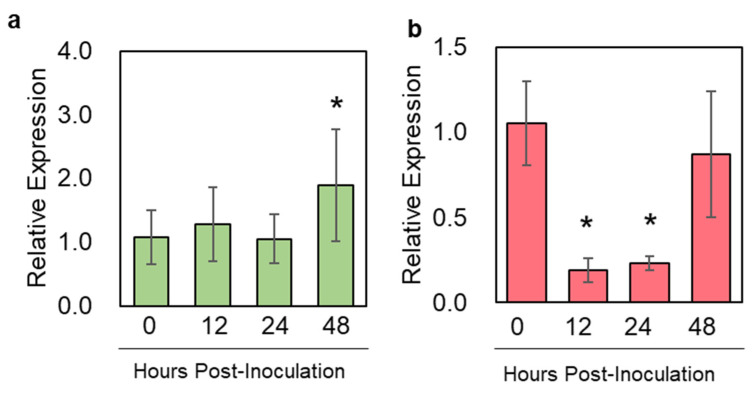
Expression of *SQUALENE SYNTHASEs* in *N. benthamiana* and *S. lycopersicum* upon *P. infestans* inoculation. Relative expression of *NbSQS* (**a**) and *SlSQS* (**b**) upon *P. infestans* inoculation. Error bars = standard deviation statistically significant differences from the mock-inoculated control group indicated by * (*p* < 0.005, Student’s *t*-tests; N = 6).

In both *N. benthamiana* and *S. lycopersicum*, the genes encoding functional SQS enzymes were characterized [34,35]. We designed paralog-specific primers to measure the expression dynamics of *NbSQS* upon *P. infestans* infection with a q-RT-PCR. In the results, the expression of *NbSQS* was significantly induced (Fold Change > 1.8) at 48 h post-inoculation (hpi) but not during earlier phases of infection (Figure 2a). In contrast, *SlSQS* expression was significantly suppressed at 12 and 24 hpi (Fold Change > 5 at both time points) and returned to its pre-inoculation level at 48 hpi (Figure 2b). These results demonstrated a host-specific transcriptional regulation of *SQS* during *P. infestans* infection.

### 3.2. Phytophthora infestans Infection Induces Transient Phytosterol Accumulation in S. lycopersicum but Not in N. benthamiana

**Figure 3:**
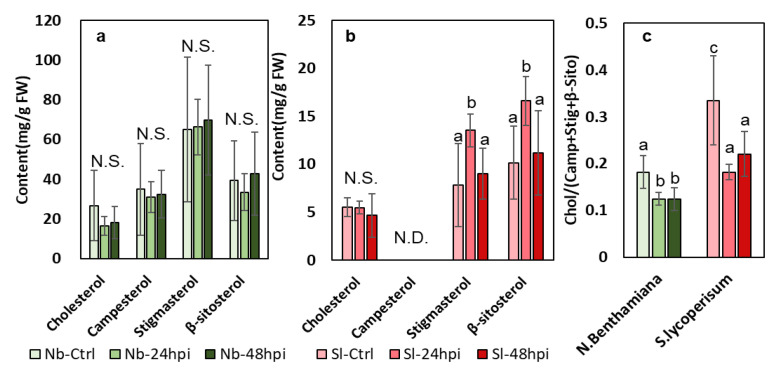
Quantification of phytosterols in *N. benthamiana* and *S. lycopersicum* after *P. infestans* inoculation. Phytosterols were measured in *N. benthamiana* (**a**) and *S. lycopersicum* (**b**) leaves at 24 and 48 h post-inoculation (hpi). Ratio between cholesterol and the sum of other phytosterols was calculated for each biological replicate (**c**). Measurements of each compound and ratio data were compared between time points with a one-way ANOVA. Significant differences are indicated by different letters on the representative columns (*p* < 0.05). Error bars = standard error; N = 5; N.S. = no significant difference; N.D. = no decetion.

In both *N. benthamiana* and *S. lycopersicum, SQS* catalyzes the production diverse phytosterols. To clarify the dynamics of the phytosterol levels during *P. infestans* infection, we measured four major compounds of this class at two different time points after zoospore inoculation. The non-host *N. benthamiana* leaves showed no significant change in any of the phytosterols upon *P. infestans* infection, whereas stigmasterol and β-sitosterol contents were significantly elevated in tomato leaves at 24 hpi (Fold Change > 1.6 for both compounds). These tomato-specific inductions appeared to be transient, as the concentrations of both compounds were reduced to pre-inoculation levels at 48 hpi (Figure 3a,b). However, as a conserved and persistent effect, the ratio between cholesterol and the methylenecycloartanol-derived phytosterols (*i.e.,* campesterol, stigmasterol, and β-sitosterol) was suppressed by more than 40% in both plant species after *P. infestans* inoculation (Figure 3c).

### 3.3. Overexpression of SQUALENE SYNTHASE Leads to Lower P. infestans Resistance and Higher Stigmasterol Content in N. benthamiana

Since post-inoculation gene expression and phytosterol measurement data suggested that *SQS* could play an important role in the interaction between plants and *P. infestans*, we aim to elucidate the function of this gene with genetics evidence. Transient silencing of *SQS* with the tobacco rattle virus system led to the lethality of the meristematic tissues, disallowing downstream inoculation assays (Figure S1). Consistently, *sqs* knock-out attempts using CRISPR technology resulted in developmental defects, precluding the acquirement of homozygous, mutant plants.

**Figure 4:**
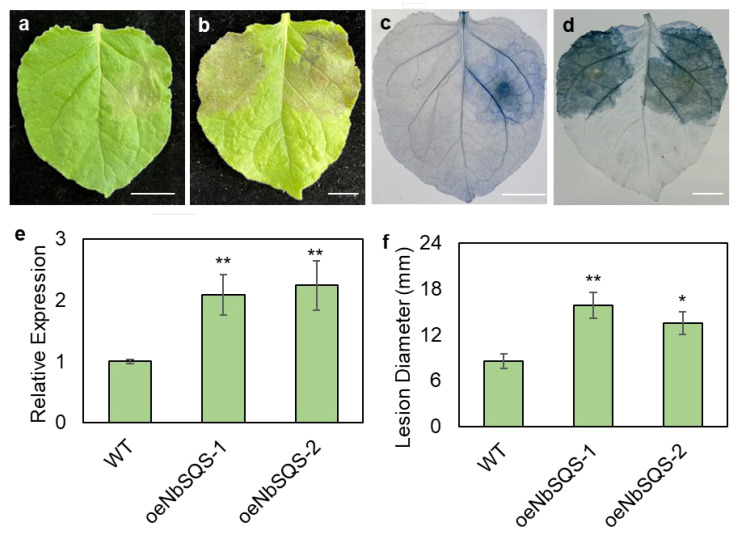
SQUALENE-SYNTHASE-overexpressing *N. benthamiana* plants are more susceptible to P. infestans. Symptoms of P. infestans inoculation on detached leaves of wild type (**a**,**c**) and oeNbSQS (**b**,**d**) plants under normal conditions (**a**,**b**) or after trypan blue staining (**c**,**d**). Scale bar (**a**–**d**) is 1 cm. Expression of NbSQS measured by q-RT-PCR; error bars = standard deviations (**e**). Lesion diameters on wild type and two independent oeNbSQS lines measured at 7 days post-inoculation; error bars = standard errors (**f**). Statistically significant differences from wild types calculated with Student’s *t*-tests (* *p* < 0.05, ** *p* < 0.01; N = 5 for q-RT-PCR; N > 24 for lesion diameter measurement).

**Figure 5:**
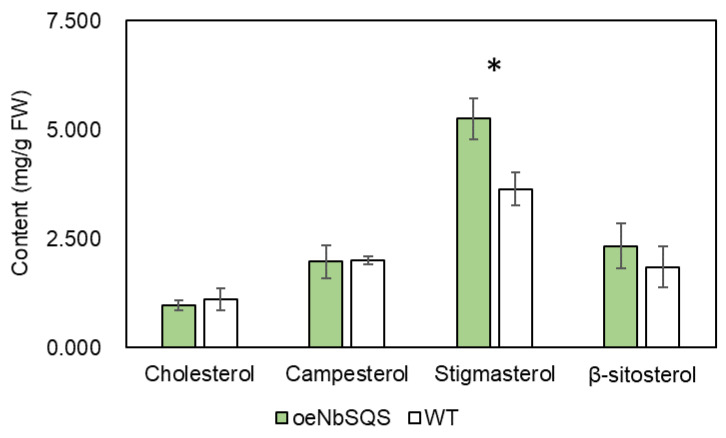
Quantification of phytosterols in wild type and *SQUALENE-SYNTHASE*-overexpressing *N. benthamiana* leaves. N = 3 for either genotype; error bars = standard deviations. * *p* < 0.05, Student’s *t*-tests.

In *N. benthamiana*, two independent *SQS* overexpression events were obtained. In these events, *SQS* expressions were elevated by more than twofold (Figure 4b). In both overexpression lines, lesion diameters were significantly increased at 7 days after *P. infestans* zoospore inoculation (Fold Change > 1.5; Figure 4a,c). We further compared the phytosterol contents in one of the overexpression lines and the wild type progenitor plants and found that stigmasterol was the only phytosterol compound significantly hyperaccumulated in the oeNbSQS plants (Fold Change > 1.4; Figure 5).

### 3.4. Transcriptomics Re-Configuration Associated with Overexpression of SQUALENE SYNTHASE

Since *SQS* encodes an important node in plant metabolism, we hypothesize that this gene can influence the plant–*P. infestans* interaction through multiple potential mechanisms. To further explore how *SQS* may affect *P. infestans* susceptibility in *N. benthamiana*, we performed comparative RNAseq analyses on wild type and oeNbSQS plants. In result, we identified 153 genes that expressed higher in oeNbSQS plants, and 121 genes that showed the opposite expression pattern. A number of these differentially expressed genes were then validated by q-RT-PCR (Figure S2).

**Figure 6:**
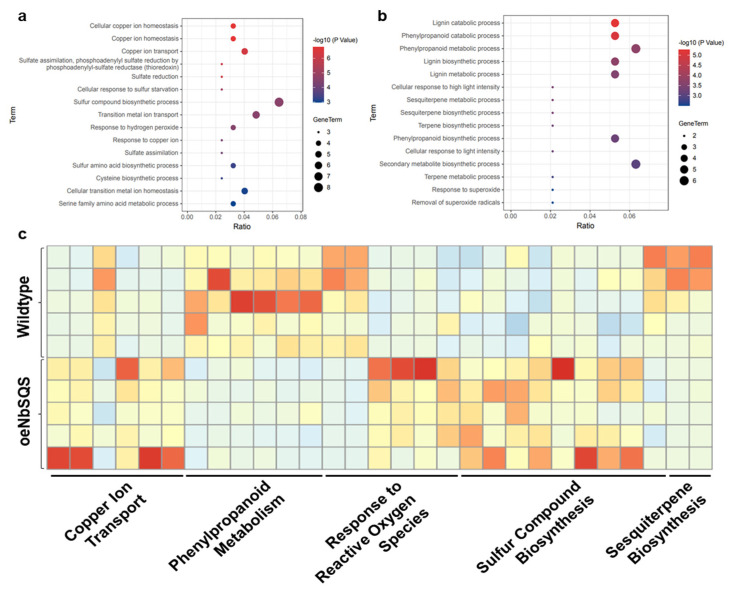
Comparative transcriptomic analysis of wild type and *SQUALENE SYNTHASE*-overexpressing *N. benthamiana* leaves. Gene ontology enrichment analysis results of up-regulated (**a**) and down-regulated (**b**) genes in oeNbSQS plants. Normalized expression heatmap of differentially expressed genes belonging to the gene ontology terms (**c**).

Gene ontology (GO) enrichment analyses showed that the NbSQS-promoted genes were disproportionally involved in sulfur compound biosynthesis (GO:0044272), copper ion transport (GO:0006825), and the response to hydrogen peroxide (GO:0042542), whereas phenylpropanoid metabolism (GO:0009698) and sesquiterpene biosynthesis (GO:0051762)-related genes tended to be suppressed when NbSQS was overexpressed (Figure 6). These disproportionally influenced pathways shed light on the multifaceted influences of *SQS* overexpression on plant physiology, some of which may mediate the impact of this gene on plant–*P. infestans* interactions.

Noticeably, we observed no significant change in the expression of brassinosteroid signaling-related genes in the oeNbSQS plants, though this pathway has been shown to suppress host immunity ([20,21]; Figure S3). This lack of transcriptional change in brassinosteroid-regulated genes suggested that the lowered *P. infestans* resistance in oeNbSQS plants was not mediated by altered brassinosteroid signaling.

## 4. Discussion

Since *Phytophthora* phytopathogens have been historically recognized to utilize plant-derived phytosterols for their own development, the manipulation of the phytosterol metabolism has been proposed as a potentially effective way to promote plant resistance against these destructive oomycetes [19]. In this study, we specifically targeted SQUALENE SYNTHASE, as this enzyme catalyzes the common precursor of all known phytosterols and triterpenoids (Figure 1). In a non-host species (*N. benthamiana*) and a host species (*S. lycopersicum*) of *P. infestans*, expressions of functional *SQSs* were subjected to differential transcriptional regulation upon zoospore inoculation. In *N. benthamiana*, *NbSQS* expression was induced by *P. infestans* only at the late phase of infection (i.e., 48 hpi), whereas *SlSQS* expression was suppressed at 12–24 h after zoospore inoculation and returned to pre-inoculation level at 48 hpi (Figure 2b,c). This expression pattern in tomatoes was consistent with results from a published RNAseq study. However, in the same study, *NbSQS* expression was shown to be significantly upregulated by *P. infestans* at 12 and 24 hpi [36]. This difference in expression pattern may be attributable to the technical difference between RNAseq and q-RT-PCR.

The lack of change in *NbSQS* expression during the initial 24 h was consistent with the stable phytosterol contents in *N. benthamiana,* whereas both *SQS* expression and phytosterol contents in *S. lycopersicum* showed transient, inducible alteration within 24 hpi. The biological significance of this correlation will require further investigation (Figure 3a,b). Similar to the findings in this study, phytosterols and triterpenoids were induced by *P. infestans* in a susceptible potato cultivar but were suppressed in a resistant line [23]. On the other hand, the conserved, *P. infestans*-induced suppression in the ratio between cholesterol- and methylenecycloartanol-derived phytosterols in *N. benthamiana* and *S. lycopersicum* appeared to be *SQS*-independent (Figure 3c). Such a shift in ratio among phytosterols could have a profound influence on plant resistance against phytopathogens and nematodes [37,38,39,40].

The differential, post-inoculation transcriptional regulation of *SQS* between a non-host and a host species indicated that this gene could influence plant resistance against *P. infestans.* To test this hypothesis, multiple genetic manipulation strategies have been deployed to elucidate the function of *SQS* in plant–*P. infestans* interactions in this study. Results of *P. infestans* inoculation experiments after transient gene silencing and overexpression were highly inconsistent across batches, which may be caused by the physical damage inflicted by the agroinfiltration operation. Furthermore, the CRISPR-mediated, stable knock-out of *SQS* in *N. benthamiana* led to a developmental defect that prevented subsequent bioassays with *P. infestans* (Figure S1). We were able to produce *SQS*-overexpressing *N. benthamiana* plants and found that these transgenic plants were more susceptible to *P. infestans* infection (Figure 4). The transient silencing of *NbSQS* was reported to compromise plant resistance against various bacterial pathogens [37]. Similarly, the silencing of *SQS* in *Withania somnifera*, a medicinal plant in the Solanaceae family, led to lowered resistance against *Botrytis cinerea* [41]. The contrasting functions of *SQS* in different plant–pathogen systems highlight the complexity of the influence of plant metabolism on plant–pathogen interactions.

To examine the physiological mechanisms that may contribute to the lowered *P. infestans* susceptibility of the oeNbSQS plants, we compared the phytosterol content between the transgenic and the wild type plants. The quantification of the four major phytosterol compounds revealed that only stigmasterol accumulated to a significantly higher level in the oeNbSQS plants (Fold Change > 1.6; Figure 5). Interestingly, this compound was also found to be significantly induced by *P. infestans* in tomatoes but not in wild type *N. benthamiana* (Fold Change > 1.4; Figure 3b). Though there has been no study on the influence of stigmasterol on *P. infestans* specifically, exogenous stigmasterol has been reported to suppress the expression of elicitin, a known, pathogen-associated molecular pattern in *P. sojae* [42]. Therefore, hyperaccumulated stigmasterol and lowered resistance against *P. infestans* in the oeNbSQS plants may be not only coincidental but also functionally associated.

Since the hyperaccumulation of stigmasterol was unlikely to fully explain the lowered *P. infestans* resistance of the oeNbSQS plants, we further explored the influence of *SQS* overexpression with comparative transcriptomic analyses. Functional enrichment analyses of the constitutively differentially expressed genes between oeNbSQS and wild type plants revealed a number of biological processes that were intuitively linked to the lower *P. infestans* resistance phenotype. For example, both sesquiterpenoids and phenylpropanoids were previously documented to enhance plant resistance against *P. infestans* [8,10,11,15]. Hence, the transcriptional suppression of genes involved in the metabolism of these classes of compounds could potentially lower *P. infestans* resistance (Figure 6). It would be interesting to confirm whether these compounds were indeed depleted in the transgenic plants with untargeted or broadly targeted metabolomics analyses in future studies. Meanwhile, the enhanced expression of sulfur-containing, compound biosynthetic genes, hydrogen-peroxide-response-related genes, and copper-ion-transport-related genes were indicative of an altered homeostasis in sulfur-containing compounds, reactive oxygen species (ROS), and copper ions (Figure 6). Recent studies have revealed that sulfur is a critical micronutrient for both plants and phytopathogens, and that plants could limit their supply of sulfur as an immune response [43]. Hence, the elevated, sulfur-containing compound biosynthesis in oeNbSQS plants may be yet another mechanistic explanation of their lowered resistance against *P. infestans*. Perturbation in ROS signaling could have a significant influence on the interaction between host plants and *P. infestans* [44]. The application of exogenous copper ions could enhance plant resistance against *P. infestans* by simultaneously promoting ethylene production and inhibiting abscisic acid biosynthesis, while apoplastic copper ions may facilitate the activity of the *P. infestans*-secreted lytic polysaccharide monooxygenases required for pathogen infection [45,46].

## 5. Conclusions

In summary, we examined the transcriptional regulation and biological function of *SQS* in the interaction between *P. infestans* and a host species, *S. lycopersicum*, and a non-host species, *N. benthamiana*. The expression of *SQS* was differentially regulated in the two plant species, and the expression patterns correlated with changes in phytosterol contents upon *P. infestans* inoculation. A stable overexpression of *SQS* in *N. benthamiana* compromised plant resistance against *P. infestans* and elevated constitutive stigmasterol contents. These two phenotypes may be functionally associated, but this hypothetical mechanism requires further investigation. Finally, the expression of a suite of genes not directly related to phytosterol and triterpenoid metabolism were found to be significantly influenced in the *SQS* overexpression plants. These genes may be indicative of additional mechanisms through which *SQS* could affect plant–*P. infestans* interactions. These results demonstrated the important function of *SQS* in plant resistance against *P. infestans and* shed light on the multifaceted impact of this gene on plant physiology.

## Data Availability

Data available in a publicly accessible repository: The data presented in this study are openly available in the NCBI SRA database at https://www.ncbi.nlm.nih.gov/bioproject/PRJNA930498/ (accessed on 8 February 2023), reference number PRJNA930498.

## Supplementary Materials

The following supporting information can be downloaded at: https://www.mdpi.com/article/10.3390/metabo13020261/s1, Figure S1: Transient VIGS of NbSQS induces meristematic lethality; Figure S2: Q-RT-PCR validation of selected differentially expressed genes identified through comparative transcriptomics analyses; Figure S3: The FPKM of brassinosteroids signalling related gene in WT and oeNbSQS lines; Table S1: List of primer sequences used in this study.
