# Supplementary material for: Overexpression of SQUALENE SYNTHASE Reduces Nicotiana benthamiana Resistance against Phytophthora infestans
Source: Metabolites. 2023 Feb 11;13(2):261. doi: 10.3390/metabo13020261 (PMC9960828; doi:10.3390/metabo13020261)
Supplement: Supplementary file 1 [file metabolites-13-00261-s001.zip › 230203-SuppFiles/230203-SQS-SuppFig.pptx]

## Slide 1
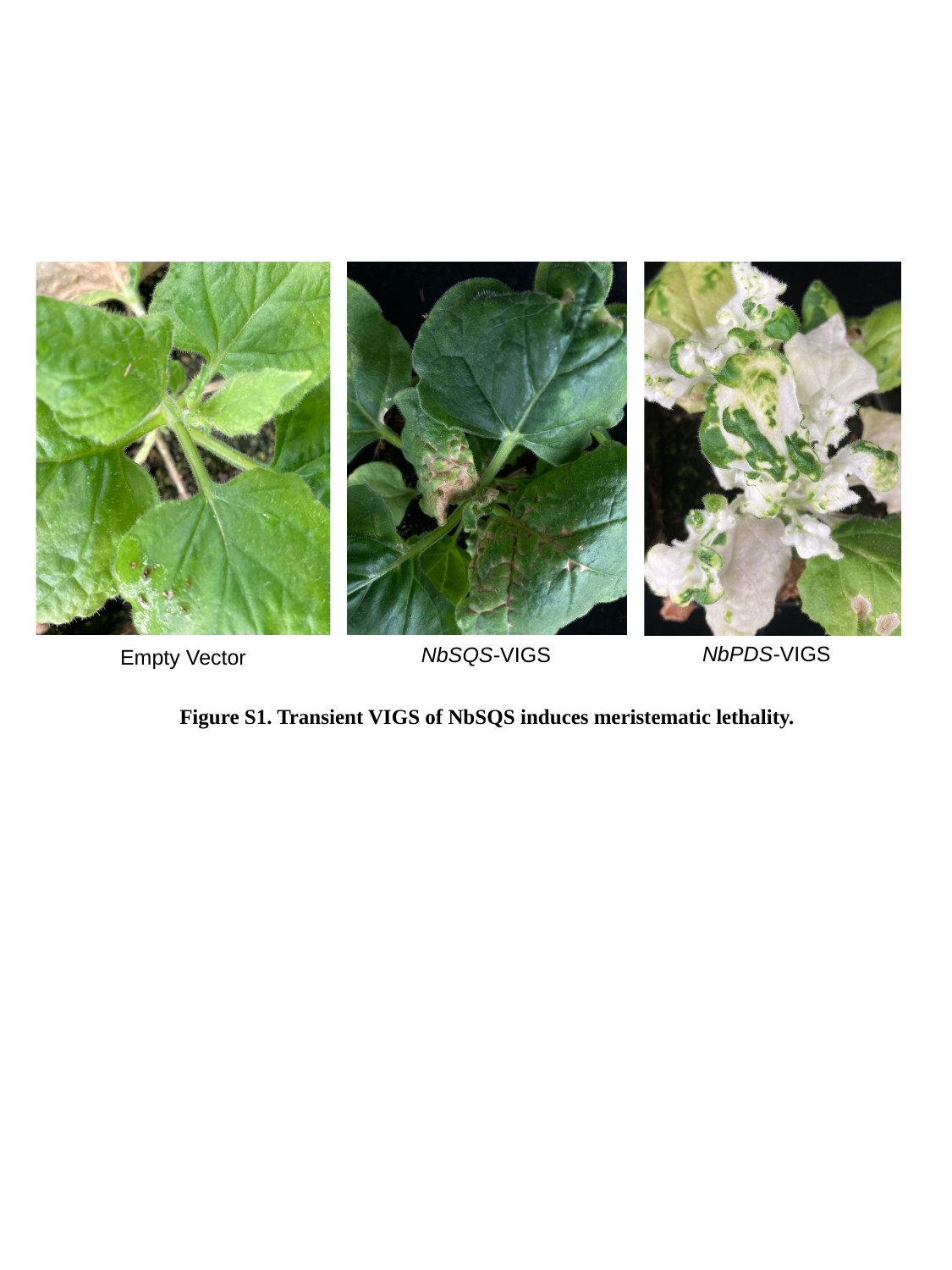

NbPDS-VIGS
NbSQS-VIGS
Empty Vector
Figure S1. Transient VIGS of NbSQS induces meristematic lethality.

## Slide 2
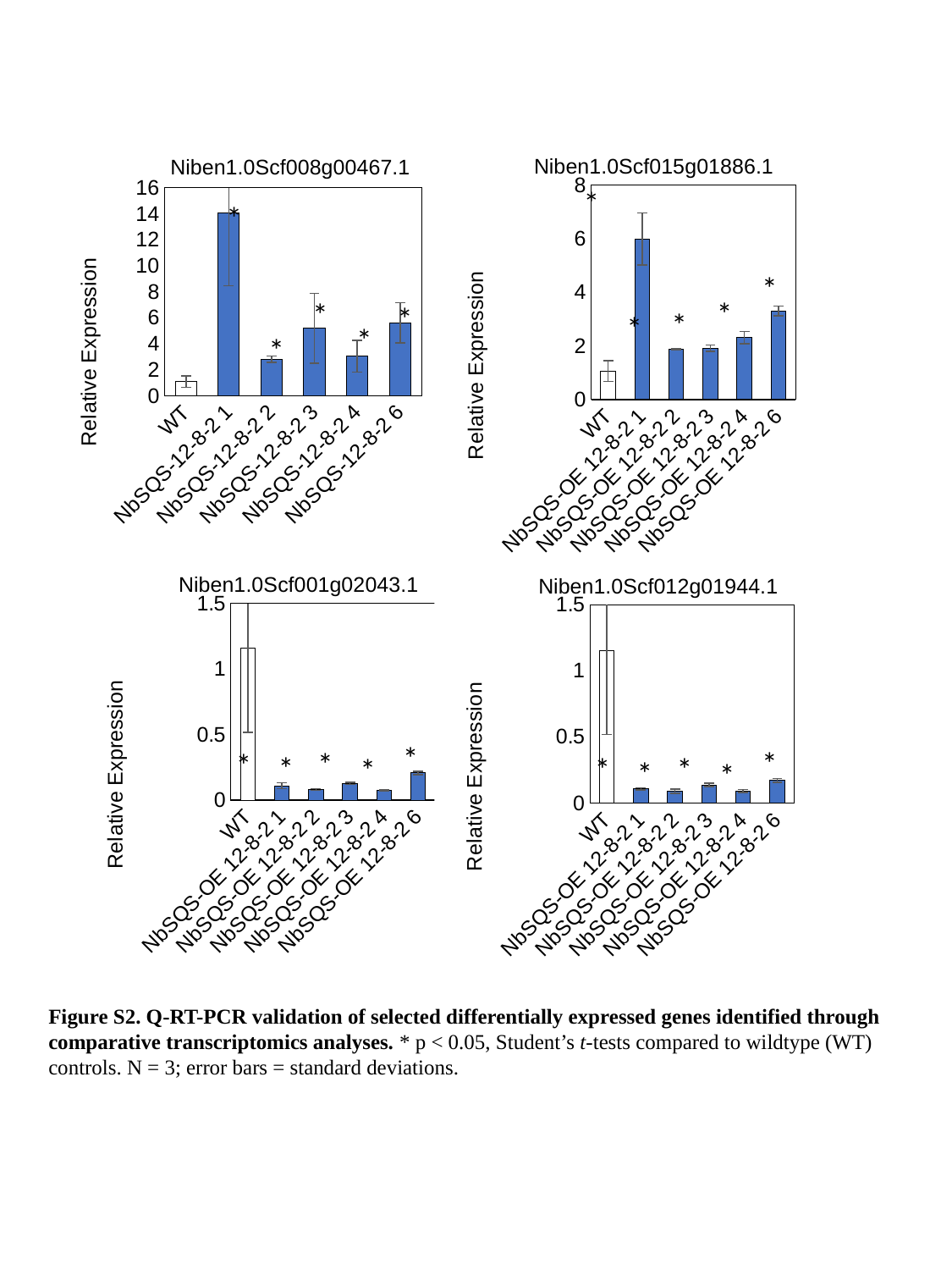

Niben1.0Scf015g01886.1
Niben1.0Scf008g00467.1
### Chart
| Category | |
|---|---|
| WT | 1.0612436098211215 |
| NbSQS-OE 12-8-2 1 | 5.986009039775741 |
| NbSQS-OE 12-8-2 2 | 1.8875575373987432 |
| NbSQS-OE 12-8-2 3 | 1.9188630451090383 |
| NbSQS-OE 12-8-2 4 | 2.3088136193973368 |
| NbSQS-OE 12-8-2 6 | 3.304332231836477 |
### Chart
| Category | |
|---|---|
| WT | 1.0809912281353247 |
| NbSQS-12-8-2 1 | 14.071637936066333 |
| NbSQS-12-8-2 2 | 2.8298806152180784 |
| NbSQS-12-8-2 3 | 5.192941030538801 |
| NbSQS-12-8-2 4 | 3.0360044504763146 |
| NbSQS-12-8-2 6 | 5.622197633526426 |*
*
*
*
*
*
*
*
*
*
Niben1.0Scf001g02043.1
Niben1.0Scf012g01944.1
### Chart
| Category | |
|---|---|
| WT | 1.159767841 |
| NbSQS-OE 12-8-2 1 | 0.108953038 |
| NbSQS-OE 12-8-2 2 | 0.081402015 |
| NbSQS-OE 12-8-2 3 | 0.128407524 |
| NbSQS-OE 12-8-2 4 | 0.075857586 |
| NbSQS-OE 12-8-2 6 | 0.208481257 |
### Chart
| Category | |
|---|---|
| WT | 1.1505494137471723 |
| NbSQS-OE 12-8-2 1 | 0.1068708271960414 |
| NbSQS-OE 12-8-2 2 | 0.0886368834083943 |
| NbSQS-OE 12-8-2 3 | 0.13760439145388978 |
| NbSQS-OE 12-8-2 4 | 0.09278020826872252 |
| NbSQS-OE 12-8-2 6 | 0.17154932965391487 |*
*
*
*
*
*
*
*
*
*
Figure S2. Q-RT-PCR validation of selected differentially expressed genes identified through comparative transcriptomics analyses. * p < 0.05, Student’s t-tests compared to wildtype (WT) controls. N = 3; error bars = standard deviations.

## Slide 3
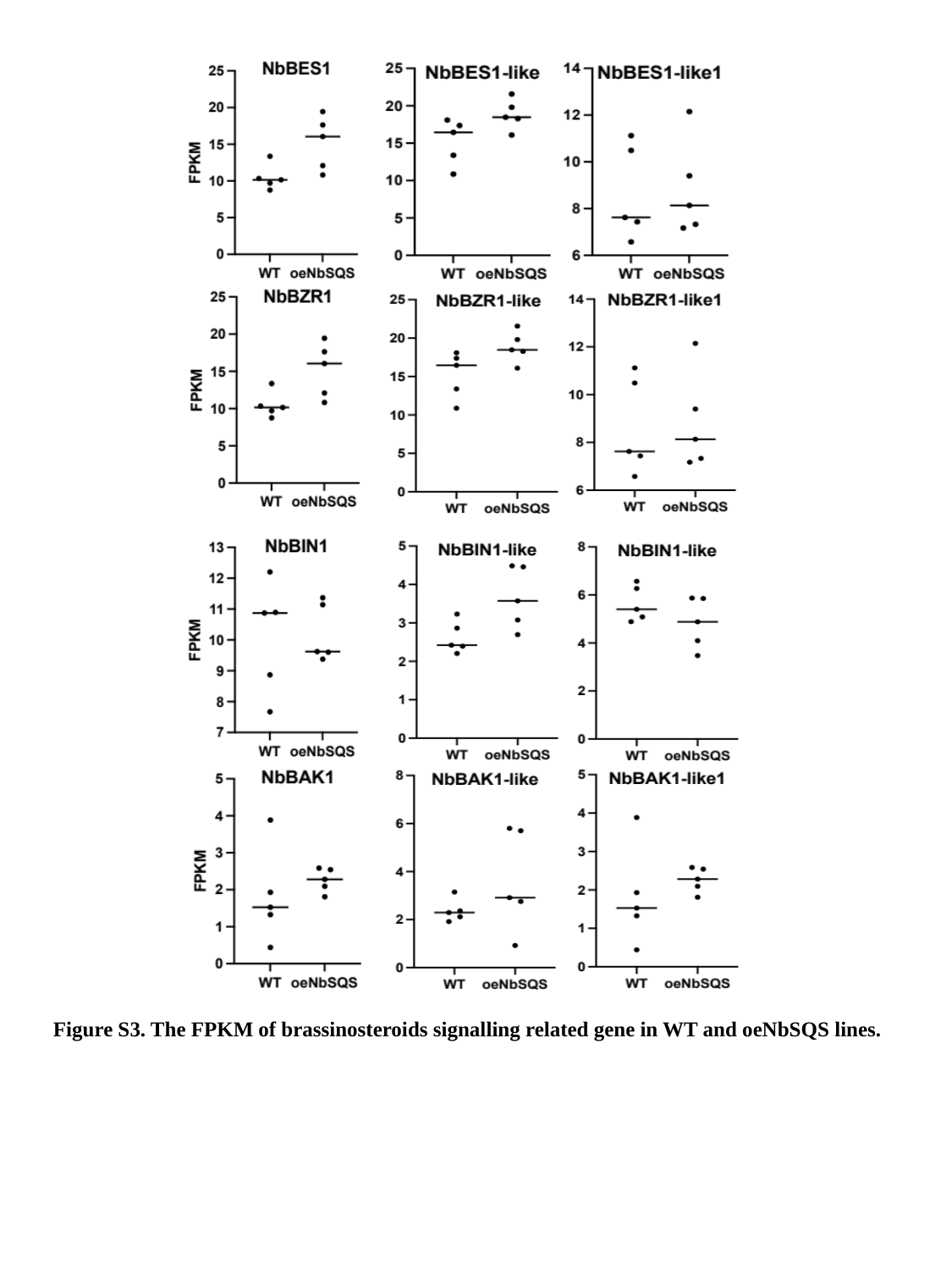

Figure S3. The FPKM of brassinosteroids signalling related gene in WT and oeNbSQS lines.
